# Letting go: Deep computational modeling insights into pH-dependent calcium affinity

**DOI:** 10.1016/j.jbc.2021.100974

**Published:** 2021-07-16

**Authors:** Matthias Buck

**Affiliations:** Department of Physiology and Biophysics, Case Western Reserve University, Cleveland, Ohio, USA

**Keywords:** lectin receptor, langerin, protein–ligand dissociation, unbinding process, endosome, molecular dynamics simulation, protein–calcium interaction, protein–carbohydrate binding, structure–function relationship, allostery, CRD, carbohydrate-recognition domain

## Abstract

Calcium and other cofactors can feature as key additions to a molecular interface, to the extent that the cofactor is completely buried in the bound state. How can such an interaction be regulated then? The answer: By facilitating a switch through an allosteric network. Although a number of unbinding mechanisms are being characterized, an extensive computational study by Joswig *et al.* reveals a detailed model for the pattern recognition receptor langerin.

“Sometimes it’s hard to let go”, is a life truth many people experience. It is also true at the molecular level when complexes between a protein and its ligand or between a protein and another protein or macromolecule need to dissociate as part of a functional cycle. The mechanisms which comprise unbinding processes have only just recently been studied in detail, in part due to the advent of advanced, if not extensive, molecular dynamics (MD) simulations (1, 2) and experimental techniques with high time/spatial resolution, such as NMR spectroscopy (3). The general questions in this field include: Is the dissociation process multistep? What are the thermodynamic driving forces? What is the level of kinetic *versus* thermodynamic control? There are likely many unbinding processes, given the great diversity of protein and (macro-)molecular interfaces. Therefore, it is a good strategy to focus on interfaces which have features in common, such as divalent cations (particularly magnesium and calcium), which are cofactors for many proteins. Both of these ions play critical roles in a vast arena of protein functions which are increasingly well characterized at the molecular level. For example, serum calcium can be sensed to exquisite accuracy by the extracellular Ca^2+^-sensing receptor, which presents a connection between Ca^2+^ binding and allosteric modulators, agonists, and antagonists for this seven transmembrane G-protein coupled receptor protein. The list of receptors of which Ca^2+^ and Mg^2+^ play a role in regulation is growing, including ryanodine, adenosine, and opiod receptors representing other recently studied cases. However, mechanisms can become even more intriguing when another common cellular component is involved. Such is the case with a transmembrane potential in mammalian Ca^2+^ and voltage-activated K^+^ channels, or for the protein at the center of this JBC Editor’s pick, the C-type lectin receptor langerin, whose carbohydrate binding is sensitive to both Ca^2+^ concentration and pH.

The C-type lectin receptor langerin is a key defense in mammals against invading pathogens. The trimeric langerin receptor on the host membrane binds to carbohydrates on the virus surface and then the langerin–virus complex is endocytosed and trafficked to an acidic endosome where it is released in a pH- and calcium-dependent manner and degraded. Despite the high level of interest in this system, the molecular details of the release mechanism have been unclear for some time. The Taylor and Weis (4) and recently the Keller and Rademacher (5) laboratories have elucidated the structural and thermodynamic as well as kinetic details of the interaction between langerin and carbohydrates at the langerin carbohydrate-recognition domain (CRD). Thus, the pH sensitivity of the Ca^2+^ affinity of langerin and other C-type lectins was discovered, but how this pH dependence occurs at the molecular level was a mystery because the Ca^2+^-binding site is covered up by the carbohydrate ligand. Furthermore, the Asp308, Glu293, and Glu285 residues directly responsible for Ca^2+^ binding have expected pK_a_ values much lower than the pH associated with the fusion with acidic endosomes (pH of 5.5–6) (5). Enter nearby His294, which was shown to titrate with a pH from outside the cell (pH ∼7) to the slightly acidic pH of the endosome. When this His was mutated to Ala there was a 40% reduction of the pH sensitivity of Ca^2+^ binding. Importantly, a crystal structure suggested that His294 forms a hydrogen bond with Lys257. However, it was unclear how His294 and/or Lys257 connect to the Ca^2+^-binding Asp/Glu residues. The report by Joswig *et al.*, 2021 (6) now proposes a molecular model based on a variety of simulations and additional experimental data.

The authors of this study used two all-atom MD simulations of the langerin CRD longer than 25 μs, one at pH 7 with His294 deprotonated and one at a lower pH with His294 protonated. The different state of His294 caused no significant perturbation to the surrounding secondary or loop structure or dynamics, but it did alter the equilibrium between several clusters of hydrogen bonding patterns between Lys257 and the Asp/Glu sidechains. These changes were not obvious but could be detected by analyzing the two conformational ensembles (langerin with protonated and unprotonated His294) in the same low-dimensional space. This space was identified by a principal component analysis on the combined MD trajectories. Careful analysis of distances and populations revealed a molecular switch involving the interaction of uncharged His294 and Lys257 at pH 7 (Fig. 1*A*). When His294 is protonated, it moves out of the way, interacting with neighboring Glu261 or Asn291. The disruption of the contact to His294 forces Lys257 to engage in new interactions. In one state of the lower pH ensemble, Lys257 interacts with Asp308, disrupting interaction with Ca^2+^ (Fig. 1*B*). In another state, Lys257 likely no longer supports an optimal positioning of Glu293, another direct Ca^2+^-binding side chain. An analysis of the trajectories in the form of a Markov model estimates that the two states described above last for 200 to 1300 ns, sufficient time for the Ca^2+^ to escape the binding pocket. Experimental measurements of Ca^2+^’s binding affinity in the His294Ala mutant suggest a residual pH sensitivity, indicating the presence of a second pH sensor. Extensive pK_a_ calculations using 10,000 to 30,000 structures extracted from these simulations suggest that in absence of Ca^2+^, Asp308 and Glu285 can form a protonated dyad with an effective pK_a_, likely high enough to sense a pH change from 6 to 7. This would amplify the protonation effect of His294. However, constant-pH simulations or quantum mechanics/molecular mechanics simulations should be run to verify this model, as the authors state (6). Although Asp308 and Glu285 are highly conserved across 15 related C-type lectins, the His294–Lys257 motif seems specific to langerin (6, 7).

**Figure 1:**
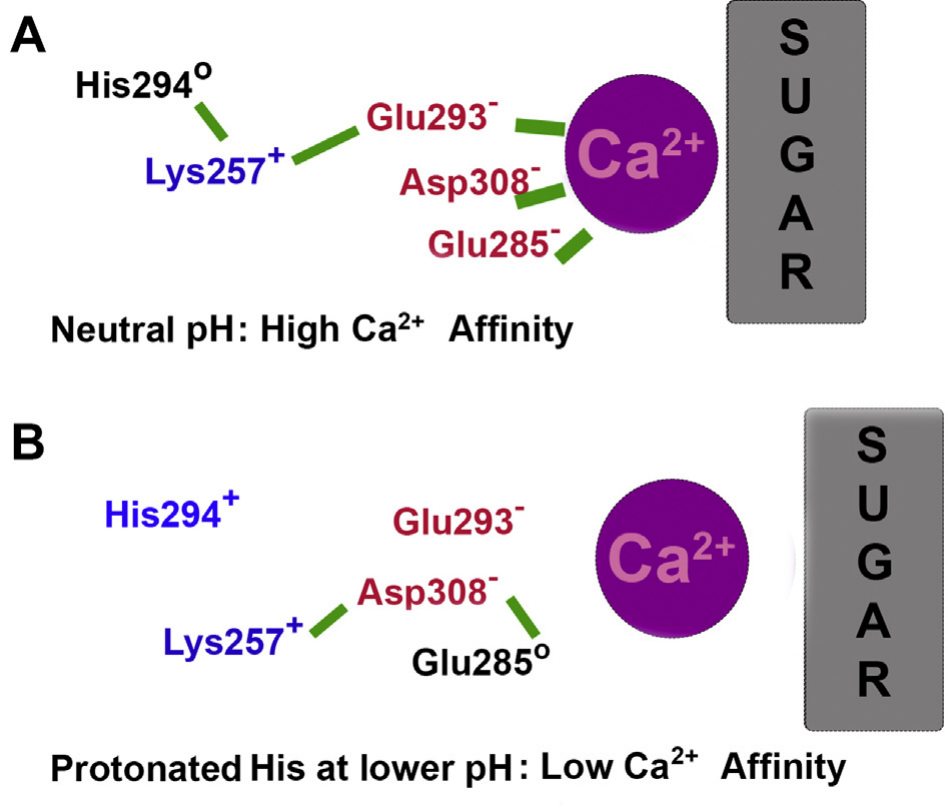
**A simplified and partial view of the mechanism described by Joswig *et al.*, 2021** (6)**.***A*, neutral pH: Network of interactions (key interactions connected by *green bars*) with high Ca^2+^ affinity. *B*, protonated His294 and possibly Glu285 lead to low Ca^2+^ affinity.

The model put forward is novel in that it derives from a subset of conformational states discovered during clustering of the MD trajectories. One concern is the lack of analysis of the role of water or solvent ions that may come in to bridge these interactions (8). Nevertheless, the model presents an elegant allosteric switch involving hydrogen-bonded and salt bridge–bonded interactions between a few residues and Ca^2+^. A follow-up study will probably be necessary to model the actual dissociation of carbohydrates and Ca^2+^ from the langerin CRD, either experimentally or using simulations.

There remain additional questions for future investigations. Early on, it was unclear how many Ca^2+^ were bound; the langerin crystal structure shows a single Ca^2+^, but other C-type lectins have up to four. Could additional Ca^2+^ play a role, perhaps as a Ca^2+^ rather than a pH sensor? Furthermore, full-length C-type lectin trimers are stabilized by a neck region that brings all three CRDs into close proximity (4, 7). Are there cooperative or other interdomain allosteric effects involved in carbohydrate release for some of these family members? The topic of avidity (9) in multivalent unbinding has recently surfaced with suggestions that such features can facilitate unbinding in the case of protein–DNA interactions (10). Whether such mechanisms are added on top of the local allosteric switch described by Joswig *et al.* (6) will be exciting to see. Typically, one needs to break several interactions, for example, move away both arms, to fully let go.

## Conflict of interest

The author declares that he has no conflict of interest with the contents of this article.
